# Adapting a Mental Health Intervention for Adolescents During the COVID-19 Pandemic: Web-Based Synchronous Focus Group Study

**DOI:** 10.2196/30293

**Published:** 2021-11-03

**Authors:** Maria Gabriela Calvo-Valderrama, Arturo Marroquín-Rivera, Erin Burn, Laura Ospina-Pinillos, Victoria Bird, Carlos Gómez-Restrepo

**Affiliations:** 1 Department of Clinical Epidemiology and Biostatistics Pontificia Universidad Javeriana Bogota Colombia; 2 Unit for Social and Community Psychiatry Institute of Population Health Sciences Queen Mary University of London London United Kingdom

**Keywords:** pandemic, COVID-19, online focus groups, qualitative research, technology, adolescents, public health

## Abstract

**Background:**

Although focus groups are a valuable qualitative research tool, face-to-face meetings may be difficult to arrange and time consuming. This challenge has been further compounded by the global COVID-19 pandemic and the subsequent lockdown and physical distancing measures implemented, which caused exceptional challenges to human activities. Online focus groups (OFGs) are an example of an alternative strategy and require further study. At present, OFGs have mostly been studied and used in high-income countries, with little information relating to their implementation in low- and middle-income countries (LMICs).

**Objective:**

The aim of this study is to share our experiences of conducting OFGs through a web conferencing service and provide recommendations for future research.

**Methods:**

As part of a broader study, OFGs were developed with adults and adolescents in Colombia during the COVID-19 pandemic. Through a convenience sampling method, we invited eligible participants via email in two different cities of Colombia to participate in OFGs conducted via Microsoft Teams. Researcher notes and discussion were used to capture participant and facilitator experiences, as well as practical considerations.

**Results:**

Technical issues were encountered, but various measures were taken to minimize them, such as using a web conferencing service that was familiar to participants, sending written instructions, and performing a trial meeting prior to the OFG. Adolescent participants, unlike their adult counterparts, were fluent in using web conferencing platforms and did not encounter technical challenges.

**Conclusions:**

OFGs have great potential in research settings, especially during the current and any future public health emergencies. It is important to keep in mind that even with the advantages that they offer, technical issues (ie, internet speed and access to technology) are major obstacles in LMICs. Further research is required and should carefully consider the appropriateness of OFGs in different settings.

## Introduction

Focus groups is a commonly used research strategy, particularly within health and social care research, where the experience of individuals, service providers, and the community is vital to innovation and implementation. Focus groups have been an essential tool of qualitative research over the last 50 years [1]; they aim to evaluate different and collective opinions of individuals in a group and pay particular attention to the interaction between participants, thus providing varied information on the topic of interest in a relatively short period [1,2]. Although focus groups offer a valuable method for eliciting a group perspective, their implementation can present a number of challenges. In particular, the need for participants to agree on a mutually convenient time and location can pose difficulties, especially in cases where the participants have existing commitments. Additionally, the recent spread of COVID-19 caused a public health emergency worldwide that affected almost all forms of human activity [3,4]. Physical distancing and lockdown during the pandemic imposed unprecedented challenges to the global population that required innovative strategies to adapt the ways people lived and worked. These recent challenges have also affected scientific research, including qualitative research methods [5], particularly projects that rely on face-to-face data collection [6].

In the past 20 years, the wide use of the internet and the availability of devices such as smartphones have enabled researchers to use web-based platforms to conduct online focus groups (OFGs). This approach has overcome some of the disadvantages of traditional focus groups [7-9]. Owing to the wide range of internet technologies, OFGs can be implemented in various ways. OFGs may be conducted using text-only platforms (ie, chatrooms, discussion boards, and emails) or as virtual group meetings by using other technologies such as webcams, smartphones, and video conferencing services [8]. OFGs can be conducted in two ways—synchronously or asynchronously, depending on whether participants share their opinions simultaneously in the platform or not. Asynchronous OFGs are generally text-based ones, where participants can answer questions through forums, email, or chat in a nonsimultaneous manner. Although the latter may provide a greater sense of anonymity, making it easier for participants to discuss sensitive topics [9,10]. An important disadvantage, however, is that researchers cannot evaluate nonverbal cues, such as eye contact, tone, and body language, that greatly enrich the results of face-to-face focus groups. Furthermore, there is a lack of interaction between participants in these settings [8]. These limitations can be overcome by using webcams in a synchronous focus group [11]. Researchers often experience a closer interaction, similar to the one obtained in a face-to-face setting. Although they tend to have less data production, the quality and level of richness of data is comparable between both settings [7,8].

The use of video conferencing software prevents the need to purchase additional recording devices, overcomes geographical barriers, and can make data transcription easier. However, incorporating new technology in research creates new methodological issues, and for OFGs to function properly, some requirements must be fulfilled. For instance, they demand participants have a minimum level of digital literacy and a stable internet connection.

These considerations can be especially challenging in low- and middle-income countries (LMICs), where the digital gap is greater as internet access is not universal; resources and infrastructure are scarce; and there is limited funding and little or no support from the government for research activities [2,12-14].

Additionally, it is the responsibility of researchers to carefully select an appropriate web conferencing service to guarantee the privacy and security of participants and the data obtained [2,7,8,15,16]. The latter is of particular significance, since privacy and security breaches have been more frequent during the COVID-19 pandemic, posing serious ethical issues to the conduct of web-based research [17,18].

Research with synchronous OFGs is a growing field; however, most of it has been developed in high-income countries. For example, Kite and Phongsavan [7] compared face-to-face focus groups with OFGs among adults in Australia; they found that OFGs produced rich data similar to face-to-face interactions and that an active discussion between participants was possible even with web-based methods. However, issues with audio, transcription and high levels of participant withdrawal were associated with the web-based modality. To the best of our knowledge, the experience of OFGs has not been reported in LMICs.

Due to the potential of OFGs to overcome the barriers associated with face-to-face methods, especially during times where physical distancing is required, it is important to evaluate their use in populations with different backgrounds. This includes vulnerable individuals and culturally diverse people living in LMICs, where OFGs offer a potentially cost-effective alternative to traditional methods. Consequently, in this paper, we aim to discuss our experiences with conducting OFGs with adults and adolescents during the COVID-19 public health emergency in Colombia.

## Methods

We developed OFGs within the framework of the BRiCs study (Building Resilience in Adolescence-Improving Quality of Life for Adolescents With Mental Health Problems in Colombia). This is an ongoing collaborative research project between Queen Mary University of London, United Kingdom, and the Pontificia Universidad Javeriana (PUJ) in Bogotá, the capital of Colombia, and Duitama, an intermediate city, with funding by the UK Research and Innovation (UKRI)—Medical Research Council. This study aims to improve health outcomes for adolescents with depression and anxiety in Colombia by adapting an existing effective app-mediated intervention called DIALOG+ [19-22]. As part of the adaptation component, 10 focus groups were planned in order to collect the end-users’ (adolescents and clinicians) and stakeholders’ (parents, guardians, youth workers, and educators) opinions, preferences, and information on how to make a resource-oriented intervention (DIALOG+) relevant in this new context and population.

In March 2020, a state of emergency was declared in Colombia due to the COVID-19 pandemic, following which the national government established mandatory quarantine and physical distancing measures. Most cities were placed in lockdown and all travel was restricted. In order to continue the research and to avoid delays concerning project deadlines, an ethics amendment was requested to change the focus group methodology by replacing the 10 face-to-face groups with synchronous OFGs performed through a secure videoconferencing system. Online delivery of the focus groups continued even when lockdown measures were eased due to the fear of contagion and advice to reduce social contact to prevent further disease spread.

Changes to the research protocol were approved by the institutional review board of both academic institutions and clinical settings (protocol FM-CIE-0084-20)

### Participants

Participants (adolescents, parents or guardians, clinicians, youth workers or teachers) were recruited from the two clinical settings in Bogotá and Duitama by using a convenience sampling method. To acknowledge the participants for their time, a Col $40 (approximately US $12 USD) grocery store voucher was offered to each participant.

Inclusion criteria for the adolescents were (1) age between 13 and 16 years; (2) self-reported current or previous experience of depression and/or anxiety; (3) a willingness to share their experience in an OFG; and (4) capacity to provide informed consent, both by themselves and by a parent or guardian. Parents or guardians were included if they provided care to adolescents aged between 13 and 16 years old with current or previous experience of anxiety and/or depression. Finally, clinicians, educators, and youth workers were (1) required to have experience working with adolescents undergoing depression and/or anxiety and (2) be at least 18 years old.

### Data Collection and Analysis

The results of this study focus on the procedures and processes involved in conducting OFGs. This includes describing, in detail, processes such as obtaining informed consent, scheduling meetings, and group facilitation, including any challenges encountered and how they were overcome. Content analysis results of the OFGs regarding the adaptation of DIALOG+ are beyond the scope of this paper and will be reported separately elsewhere.

Data for the present study were collected through participant observation, with the researchers and group facilitators taking notes during the focus groups. The notes focused on the procedures undertaken, the experience of the group facilitators, and the differences observed between web-based and face-to-face delivery. These observational notes and descriptions of the procedures were gathered by the study coordinator and were discussed during team meetings conducted after the OFG sessions. These reflexive evaluations allowed identification of issues and problems, and action was taken to find potential solutions through discussion with the research team. Additionally, content analysis of the OFG transcriptions was performed when participants expressed their opinions or thoughts related to the methodology used. A final revision of the analyzed content was performed in order to group the information into 7 different categories: consent, booking, facilitation, technical considerations, interaction, and content, which we explore below. This process enabled us to develop themes and guidance, which may be used to guide the future conduct of OFGs.

## Results

Below, we first describe the sample and then outline the procedures involved in conducting the OFGs, including any challenges faced.

### Sample

A total of 10 OFGs were conducted. In all, 47 participants were approached and only 2 did not participate, which is not unlike the withdrawal rate expected for face-to-face focus groups. One participant did not respond to the invitation email, and the other was unable to participate due to personal circumstances. With a total of 45 participants, each OFG comprised 3 to 7 participants. Participants joined the OFGs mainly from their homes and workplaces. Most of the participants used laptops and desktop computers as their primary device, with a minority using smartphones. Interestingly, tablet devices were not used by the study participants. Table 1 describes the number and general characteristics of participants in each OFG.

**Table 1:** Participants in each online focus group.

Group			Female (n)		Male (n)
**Adolescents**					
	Bogotá (first)	2		3	
	Bogotá (second)	3		2	
	Duitama	3		0	
**Parents or guardians**					
	Bogotá	3		0	
	Duitama	3		0	
**Clinicians**					
	Duitama (first)	1		4	
	Duitama (second)	3		1	
	Bogotá	5		0	
**Youth workers or teachers**					
	Bogotá	4		3	
	Duitama	5		0	

### OFG Procedures

#### Obtaining Informed Consent

The study coordinator invited the participants via email. In the case of parents and adolescents, the invitation email was followed by a phone call. The email and the follow-up phone call provided participants with the relevant information about the study and explained the role of each participant, possible risks, and other information as required. If participants were interested, informed consent was obtained remotely, and individuals were asked to complete a sociodemographic questionnaire. For adolescents, an additional invitation letter explaining the project was sent to their parent or guardian, and we verified that their informed consent had both the adolescent’s and guardian’s signatures.

As receiving an ink signature for informed consent was challenging due to the circumstances, we obtained an electronic signature from all participants. We also retrieved a signature from the parents or guardians of the participating adolescents. To obtain the electronic signatures, participants were requested to print and sign the informed consent form and send the scanned file to the study coordinator, who was in charge of verifying that every participant had properly filled and sent it prior to each session. All signatures were obtained without difficulty, and none of the participants required assistance or had doubts regarding the process.

#### Procedure

After obtaining informed consent from the study participants, the study coordinator sent separate invitations for two different meetings. The first meeting included a trial run to check the participant’s internet connection and to confirm that all the participants were able to join the OFG and use the videoconferencing software without difficulties. Additionally, during this first trial meeting, the coordinator explained further details of the project and solved logistic and participation queries. The second invitation was for the OFG session. For both invitations, the time, date, and agenda were included.

#### Booking

Overall, scheduling the focus groups was not problematic. When sending the invitations to the potential participants, an initial date and time (ie, hour) was stipulated by the study coordinator. We did, however, experience an issue with one OFG session with clinicians—the invitation for a session that was scheduled in the morning (AM) was mistakenly sent for the evening (PM) due to a typographical error. Fortunately, one of the participants double-checked this with the coordinator, and the mistake was rectified in time to enable the rest of participants to join the meeting at the correct time.

### Participants’ and Researchers’ Experience

#### Facilitation of Focus Groups

Each OFG was facilitated by 2 core members of the research team (LOP and CGR who are psychiatrists and academic researchers) and an anthropologist, who have extensive experience conducting focus groups. Decisions regarding who would facilitate each OFG was based on availability of the team members. Within the group, we allowed multiple people to speak at the same time, to keep the dynamics similar to that of a face-to-face group; however, using the “raising of hands” function on Microsoft Teams was encouraged.

Initially, the facilitator introduced him or herself, provided a general description of the team, and shared the expectations for the session as well as the ground rules (see Textbox 1). We then asked if every participant had read and understood the informed consent and checked that everyone agreed with recording the session (audio backup recording was also in place). We reminded participants that the audio would be transcribed without any identifying data, so anonymity was ensured, and requested both participants and researchers to activate their web cameras in order to obtain visual cues. Each participant was then asked to provide a brief introduction.

Facilitators followed session guidelines so that the topics for discussion were consistent in all OFGs. An observer was off camera, taking notes of the visual cues and the process. All OFGs were conducted within 90 to 120 minutes. In general, the facilitator’s role in OFG was more active than in a face-to-face scenario, both for encouraging and moderating participation, as well as maintaining order to avoid simultaneous speaking when a discussion was ongoing.

**Ground rules as per the facilitators’ guidelines.**

Ground rules:

- Properly introduce yourself and the team. Clarify the purpose of the session and of the data collected.
- Remind participants that the sessions will be audio- and video-recorded and transcribed.
- Explain how confidentiality is ensured.
- Emphasize that there are no right or wrong answers, just points of view and opinions on the DIALOG+ intervention.
- Suggest participants to avoid naming institutions or people when talking about their own experiences, but if they do, remark that it will be erased from the transcription.
- Participants may use a pseudonym.
- Ask participants to speak one at a time to avoid interrupting others.
- Clarify the duration of the session (120 min).
- Remind participants that they can leave or take a break at any point during the discussion.

#### Technical Considerations

Currently, there are several web conferencing services available to facilitate video calls. To select the best one, we evaluated different options and asked the Information and Communication Technology Service of PUJ for advice. The main criteria included finding a platform that prioritizes security and data privacy. We completed the first 3 OFGs in real time using the web-based platform Cisco Webex [23]. During these first OFGs with clinicians, the initial part of the session was spent resolving issues and concerns related to the platform, such as how to join the meeting and activation of the camera and audio, which made communication slower. Participants mentioned that they felt the video conferencing was not as easy as they expected because they were not familiar with the interface, which is not widely used within Colombia. Therefore, we decided to host the remaining OFGs with Microsoft Teams [24], a technology that is more commonly used within Colombia.

In order to overcome this challenge and familiarize participants with the platform, we sent an instructions manual, via email, explaining how to set up a Microsoft Teams account and join the virtual meeting. To further assist the participants, we scheduled a short meeting with the study coordinator before each OFG, to test connectivity and solve technical issues. As suggested by Kite and Phongsavan [7], we also encouraged early login to the platform on the day of the session [7]. Nonetheless, most participants joined the meeting a few minutes after the stipulated time.

When using Microsoft Teams, we noticed that joining the meeting was easier for those participants who had the desktop app installed on their computers than for those using the browser version. We therefore recommended installing the app prior to the OFG. However, we did experience issues with the audio during one OFG session. After the participant changed his microphone and restarted the software on his computer without success, we suggested using the browser version that solved the issue.

In general, all of these measures undertaken helped us to utilize most of the scheduled time for each session with discussion relevant to the research, rather than with technical discussion; it also made participants more involved, even if personal matters, including children, pets, and phones, would sometimes distract them momentarily during participation from their homes.

As researchers, organizing conventional face-to-face focus groups is challenging, especially in large urban areas such as Bogotá; hence, OFGs were perceived as a good alternative. In a smaller city such as Duitama, on the other hand, we had concerns such as less stable internet connection. However, network coverage was better than anticipated. Therefore, this dismissed our concern regarding internet connectivity and device availability.

As expected with adolescents (both in Bogotá and Duitama), we did not face any technical challenges, and participants’ use of the web conferencing service was seamless. In the test meeting, we had no discussion about the use of the platform, and only general concerns about the informed consent form and sociodemographic questionnaire were addressed.

Adult participants in Duitama seemed to face more challenges with using the web conferencing service, and they had more queries about the platform, which needed to be resolved. It is possible that individuals in Duitama, which is an intermediate city, were not as familiar with conferencing services as those within major urban areas such as Bogotá. Since both settings were urban areas, we did not encounter problems with internet connection. Moreover, other challenges, such as those pertaining to audio quality, as reported previously [7,25], were not a major issue in our study, which made audio transcription easy. All sessions were recorded using web conferencing recording features, and a backup audio recording was made by the study coordinator using the computer’s audio recorder.

#### Participation Interaction

All participants agreed to activate their cameras, this enabled us to ensure similar interactions to that expected in a face-to-face focus group. Questions were presented following a predetermined order, from general to more specific topics according to a previously developed facilitator’s guide. Raising hand via emoji within the software or physically by the participants raising their hand via the camera allowed participants to take turns. Although sometimes participants tended to speak simultaneously making it difficult to hear all opinions, it was the duty of the facilitators to remind individuals to take turns as would happen in a traditional face-to-face group.

The facilitator encouraged all participants to share their opinions on each topic presented. Participation was modest during the initial parts of every OFG, which can also be common in face-to-face focus groups [26]. We noticed that after a few questions, engagement increased, and participation began to be spontaneous.

This modality lacked features such as small chats and paired discussions between participants. It was obvious that participants who knew each other before the OFG (eg, clinicians and teachers and youth workers) were more engaged and participated more than those who did not know each other beforehand. Spontaneous social interaction and acquaintance between unfamiliar participants did not happen during the groups. Given that the online context can overlook some nonverbal cues, the facilitators had to rely on asking direct questions to invite people to share opinions and make sure that everyone could share their view without interruption.

Overall, the feedback obtained suggested that both researchers and participants perceived OFGs as a good alternative to face-to-face groups. Particular logistical advantages were discussed. In both settings, particularly in Bogotá, OFGs were perceived as less time consuming because there was no need to factor in travel time, which in a large city or rural area can be significant.

Additionally, costs were diminished because we did not have to consider transportation fees, hospitality, or additional recording equipment because all sessions were recorded through the recording features of the web conferencing platform.

#### Content of the Focus Groups

Our project discussed an app (DIALOG+) aimed to improve outcomes of depression and anxiety in adolescents based in Colombia. As mentioned, participating adolescents had self-reported current or previous experience with anxiety and/or depression, and all stakeholders had experience in this field. Discussing mental health has the potential to open up sensitive topics that can trigger distressing responses from participants, particularly adolescents, who may require additional support. We did not experience this issue in our study, as none of the participants reported feeling distressed by the topics discussed.

However, we consider that for all focus groups, regardless of the modality (ie, face-to-face or web-based focus groups), this aspect must be considered when sensitive topics are discussed as part of the study. Strategies to manage participant distress should be discussed between researchers, such as providing additional resources (eg, helpline and crisis contacts), clinical staff on site, or appropriate referral pathways.

In our case, 2 of the facilitators (CG and LOP) were also clinicians with extensive experience in child and adolescent mental health, and they were available to be contacted during or after the session if a participant required help, in which case they would evaluate the need for treatment or additional interventions and refer them to the appropriate services.

## Discussion

During unprecedented circumstances, OFGs were a useful tool to guarantee research continuity in cases where physical distancing was mandatory [27]. This change of methodology generated new knowledge and skills for the members of our research team and enabled us to reduce significant delays in meeting our research deadlines as well as collect considerable data that allowed us to fulfill the aim of this phase of our research project. Furthermore, the ease of organizing and scheduling OFGs, especially for clinicians and individuals who lived in larger geographical areas, offered a viable alternative to face-to-face meetings. However, with the introduction of a new technology to a traditional research method, we expected new challenges. Based on our experience conducting OFGs with multiple stakeholders, a summary of our recommendations on performing OFGs is shown in Textbox 2.

**Summary of recommendations to perform OFG.**

**Before the online focus group:**

- Verify dates and hours scheduled. Double-check for any typographical errors or autocorrections in invitations.
- Consider several web conferencing services. Consider privacy and security settings, as well as familiarity of participants according to local contexts.
- Schedule a trial meeting of short duration before the actual online focus group session to check participant’s internet connection and solve any doubts.
- Send a brief instructive email explaining how to join the meeting, activate audio and camera, and install the selected platform desktop version.
- Consider incentives to minimize possible withdrawals.

**During the online focus group:**

- Encourage early login and use of headphones with built in microphone, if available.
- Schedule more time than you would for a face-to-face focus group. This allows facilitators to perform ice breaker activities and deal with technical issues that may arise.
- Fewer participants (between 4 and 6) may have more interaction and active participation than larger groups.

The majority of challenges faced were related to technology literacy and a lack of familiarity with new videoconferencing software. While selecting the platform to conduct OFGs, it is important to consider which web conferencing platform is the most suitable for the particular target population [7,8]. We suggest considering the security and privacy settings offered by each one, as well as the familiarity participants might have with them [11]. Research suggests that participants tend to be less distracted by the web-based platform when they are more familiar with it [15]. We noticed less discussion regarding technical issues when we used Microsoft Teams compared to Cisco Webex, maybe because the former is more commonly used in Colombia. Therefore, when considering which platform to use for OFGs, it is important to consider the preference and familiarity of the target population.

To increase participant familiarity with the videoconferencing system, particularly for those that had not used it before, sending instructions on how to join meetings and setting up the microphone and web camera can be useful, as well as prompting individuals to install the desktop app. For the latter, verifying that the application can access the microphone and camera is vital (especially on Windows PCs). Prior to conducting a focus group (both face-to-face and OFGs), we recommend participants to double-check dates and hours on invitations, pay special attention to any typographical errors, or autocorrections that might have occurred.

Another strategy that helped us to use the time of the OFG session for research-related topics was performing a trial meeting the day before to solve concerns (related to the web platform or the research itself), provide more personalized assistance, and encourage participants to login early on the day of the session. Despite specific instructions and the test trial, minor technical issues still occurred. However, overall, these measures considerably minimized technical and procedural issues. Since technical challenges cannot be prevented in their totality, we consider that it is better to schedule more time for an OFG than you would for a face-to-face focus group [7]. Allowing additional time may also help because the novelty of the modality can cause participants to be initially apprehensive about participating in the discussion. Therefore, additional time can be used to implement strategies that stimulate contribution from each participant, such as introductory and ice-breaker activities.

Since both settings of our study (Bogotá and Duitama) were urban, access to computers and a reliable internet connection were available and made it easier for us to conduct the research. If we were to conduct OFGs in rural areas, it is likely that we would have faced greater challenges, especially in an LMIC like Colombia.

Unlike the experiences reported by Tuttas [25] and Kite and Phongsavan [7], we did not face any challenges regarding sound quality. Most participants were using headphones with built-in microphones, so we were able to allow all participants to be unmuted, thus allowing a more fluent discussion without much background noise. This also meant that we did not face any problems with the audio transcription.

We paid particular attention to the aspect of informed consent, especially since the study involved adolescent participants. All participants received the informed consent forms electronically and were able to seamlessly send a scanned version with their signatures, as well as their parent’s or guardians’ signature, as required. This avoided any form of face-to-face contact with the participants during the COVID-19 pandemic.

Another factor that must be kept in mind, is the number or participants that is optimal for OFGs. In our experience, we consider that the optimum number of participants for OFGs would be between 3 and 5. This number is fewer than would be expected in a traditional face-to-face focus group [28,29]. Small groups were preferred as they facilitated more interaction. When the number of participants was higher, we observed that some participants answered most questions and the rest would rarely speak (unless directly asked), or the group interactions were less extensive.

We consider that the skills of the facilitator are crucial for directing OFGs. Certain nonverbal cues are visible via the webcam (eg, head nodding or raising of hands), but others, such as direct eye contact that can signal eagerness to speak, are lost. Therefore, interaction has to be more direct. The facilitator has to actively invite people to communicate and has to be tactful when there are multiple interactions, allowing everyone to express their opinion without interrupting abruptly. With this in mind, explaining ground rules on participation at the beginning of the session is of great importance.

Previous studies have shown higher rates of dropouts [11,15] and withdrawal in OFG, especially when there is a discussion of sensitive topics [25]. We did not experience this, probably due to the nature of the topic discussed, and the incentive of a receiving store voucher could also have minimized dropouts. Considering these kinds of incentives might help reduce the rates of withdrawal. Another factor that could have contributed is that these OFGs were conducted during lockdown and the consequent physical distancing measures. Therefore, most of our participants were working or studying from home and probably had more availability to participate than in another setting.

It is important to highlight an aspect of our research, which is developing OFGs with adolescents. The use of the internet in this age group can be seen as a less intimidating way of encouraging participation [9], and their familiarity with the online world gives them an augmented sense of control. OFGs were perceived as a less hierarchical interaction than the one in a face-to-face contact [30]. In our case, adolescents were clearly fluent in the use of the web conferencing software, and we noticed that participants, across all groups, actively participated in both study sites.

### Limitations

The major limitation of this study was that given the current situation, the quality of the information obtained with the OFGs could not be compared to that obtained from a face-to-face group. This means that we do not know if the quality of data was significantly different. When using a familiar web conferencing service, participants mentioned feeling comfortable with the methodology and stated that they expressed their opinions without feeling restricted by the form of communication. Therefore, we consider that the quality of information obtained would have been similar had we conducted a face-to-face focus group. We believe that sharing our experience of conducting OFGs with adolescents in an LMIC, in the context of the COVID-19 crisis, can be valuable for future researchers.

### Conclusions

Overall, our experience using web conferencing services to perform OFGs was successful.

We consider that the current technological advancements provide OFGs with great potential in research settings, especially in the current global pandemic that has made it difficult to conduct research. A positive aspect of the current pandemic may be that a greater number of people who were unlikely to use web conferencing services are now familiar with them.

It is important to note that even if this modality can overcome geographical barriers, technical issues such as internet speed and access to equipment are great obstacles in LMICs, especially in rural areas. The access and knowledge to these platforms reflect a level of access to technological resources that is not yet universal, which means that many groups can be underrepresented [2]. For example, certain groups, such as the elderly or those from lower socioeconomic backgrounds, may lack access to technology and/or technical competencies required.

For the purposes of our research, OFGs allowed continuity with satisfactory results, and the objectives for the initial stage of our study were met thanks to the quality of data obtained. Further testing of this method is required to overcome current limitations.

## Abbreviations

BRiCs: Building Resilience in Adolescence-Improving Quality of Life for Adolescents With Mental Health Problems in Colombia
LMICs: low- and middle-income countries
OFG: online focus group
PUJ: Pontificia Universidad Javeriana
UKRI: UK Research and Innovation
